# An Unusual Presentation of Adult Intussusception

**DOI:** 10.7759/cureus.55501

**Published:** 2024-03-04

**Authors:** Hannah Z Weiss, Aidan Snell, Brandon W Knopp, Jeniel Parmar

**Affiliations:** 1 Medical School, Florida Atlantic University Charles E. Schmidt College of Medicine, Boca Raton, USA; 2 Endocrinology, Florida Atlantic University Charles E. Schmidt College of Medicine, Boca Raton, USA; 3 Emergency Medicine, Florida Atlantic University Charles E. Schmidt College of Medicine, Boca Raton, USA

**Keywords:** heart block, bradycardia, abdominal pain, colonic adenocarcinoma, right hemicolectomy, intussusception

## Abstract

Intussusception is a condition characterized by the invagination of a proximal segment of the intestine into a distal segment. In adults, intussusception is commonly associated with a lead point. The most alarming lead point is an obstructing malignancy. Here, we present the case of a 57-year-old woman with ileocolic intussusception secondary to colonic adenocarcinoma. The patient presented to the emergency department following an incidental finding of bradycardia, with a heart rate of around 40 beats per minute. She presented with several weeks of cramping, right lower quadrant abdominal pain, lightheadedness, fatigue, and palpitations. A computed tomography scan revealed ileocolic intussusception. After the placement of a semi-permanent right subclavian pacer, the patient underwent a right hemicolectomy. Surgical findings were consistent with ileocolic intussusception suspicious of being initiated by a mass in the right cecum involving the appendiceal orifice and ileocecal valve that invaded through the muscularis propria into subserosal tissue. The mass was resected and sent to pathology, where it was classified as stage II colonic adenocarcinoma. This case highlights a nonspecific presentation of intussusception that was only identified due to incidental bradycardia.

## Introduction

Intussusception is the telescoping of a proximal bowel segment into a more distal bowel segment. The condition is far more prevalent in children, with only 5% of reported intussusceptions occurring in adults [1]. While pediatric cases of intussusceptions are typically idiopathic, 75% to 90% of adult intussusceptions have an identifiable lead point [2]. Benign etiologies, such as abdominal adhesions and strictures, may act as lead points for adult intussusception. Unfortunately, malignant lesions are the most frequently identified lead points. In adults, an estimated 66% of colonic intussusceptions and 30% of small bowel intussusceptions are attributed to malignant lesions [3].

Adult cases of intussusception are difficult to diagnose due to their rarity, with only 1-2 reported cases per million of the adult population per year, as well as their varying presentations [4]. Symptoms in adult cases of intussusception are nonspecific, ranging from generalized cramping abdominal pain, nausea, and diarrhea to emesis, blood per rectum, and constipation [5]. Consequently, the most frequently cited symptoms are those of a small bowel obstruction. In the past, only 40-50% of adult intussusception cases were diagnosed preoperatively [1]. Fortunately, advancements in computed tomography (CT) continue to increase the rate of preoperative intussusception diagnosis. Early diagnosis and management of intussusception in adults are crucial to prevent complications, such as bowel ischemia and necrosis from obstruction. Here, we present the case of a 57-year-old female with intussusception secondary to colon adenocarcinoma.

## Case presentation

A 57-year-old woman with no significant past medical history presented to the emergency department following an incidental finding of bradycardia, with a heart rate of around 40 beats per minute. She presented with several weeks of cramping in her right lower quadrant, lightheadedness, fatigue, and palpitations. The patient denied chest pain, shortness of breath, syncope, hematuria, or melena.

On examination, the patient was not in acute distress. The patient presented with a body mass index of 16 kg/m². Her vitals included a temperature of 36.5°C, a heart rate of 58 beats per minute, a respiratory rate of 11 breaths per minute, and a blood pressure of 128/58 mmHg. Physical examination was significant for right lower quadrant abdominal tenderness without rebound or guarding. No masses were palpated on the abdominal or rectal examination. The cardiovascular examination revealed an irregular rate and rhythm. The patient was started on an isoproterenol drip after an echocardiogram revealed a 2-1 heart block alternating with a complete heart block and accelerated junctional rhythm.

**Figure 1:**
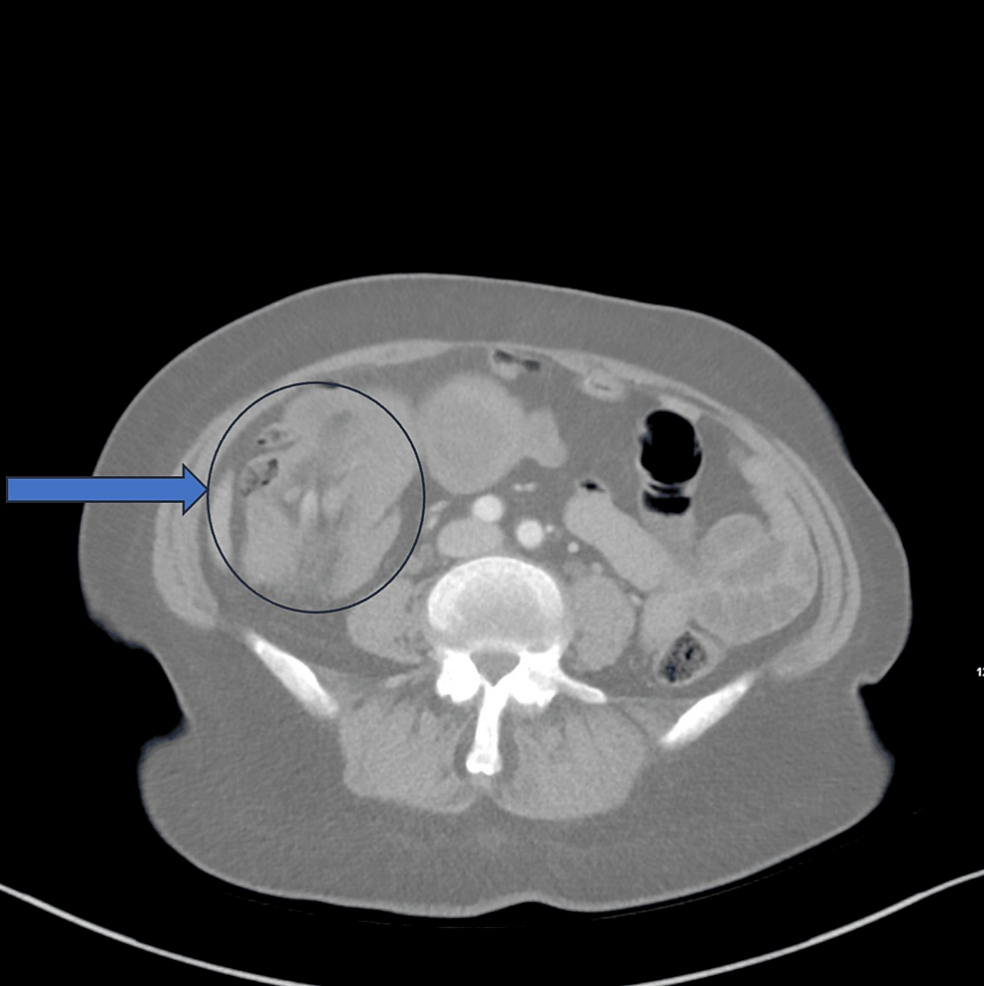
Computed tomography scan illustrating intussusception. The patient’s intussusception is seen with the characteristic “doughnut” sign encircled above.

The patient’s blood work revealed a red cell count of 2.41 x 10^6^/µL and a hemoglobin of 3.9 g/dL. After receiving two units of packed red blood cells (PRBCs), the patient’s hemoglobin increased to 7.8 g/dL. A CT scan revealed ileocolic intussusception (Figure 1). The patient was admitted to the stepdown unit and was scheduled for a right hemicolectomy the following day. Overnight, the patient was transferred to the intensive care unit after her heart rate slowed to 30-35 beats per minute with episodes of complete heart block.

The following day, the patient was transfused an additional unit of PRBCs after her hemoglobin decreased to 6.9 g/dL. She was subsequently started on sodium ferric gluconate complex for three days. The patient was also seen by an electrophysiologist, who recommended the placement of a semi-permanent right subclavian pacer before surgery. After placement of the semi-permanent right subclavian pacer later that day, isoproterenol was discontinued, and the patient underwent an open right hemicolectomy. Surgical findings were consistent with ileocolic intussusception suspicious of being initiated by a mass in the right cecum involving the appendiceal orifice and ileocecal valve that invaded through the muscularis propria into subserosal tissue. The tumor was 5.9 cm in the greatest dimension, polypoid, infiltrative, and ulcerated. Fourteen lymph nodes were also resected and sent to pathology. A right colon specimen containing the tumor was sent to pathology, which revealed stage II grade II colonic adenocarcinoma with mucinous features and negative margins. The resected lymph nodes were negative for carcinoma involvement.

After hemicolectomy, the patient was started on glycopyrrolate and monitored for possible placement of a permanent pacemaker. On postoperative day five, the patient’s rhythm was determined to be a complete heart block, and a permanent pacemaker was placed. The patient recovered without complications and was discharged seven days after hemicolectomy. Oncology determined that the tumor was likely sporadic and did not necessitate adjuvant treatment or radiation. The patient was scheduled for observation via CT scan every six months.

## Discussion

Early diagnosis of adult intussusception should be prioritized due to the association of the condition with malignancies. Rapid diagnosis of intussusception is particularly critical in adults as treatment is almost always operative. Delays in treatment are associated with significant morbidity and mortality. The patient discussed above had experienced several weeks of nonspecific right lower abdominal pain but did not seek treatment for this symptom.

If the patient did not present with bradycardia, then her intussusception and colonic adenocarcinoma would have likely gone undiagnosed. Although the bradycardia may have been vagally mediated, the persistence of heart block after the resolution of the intussusception suggests that the bradycardia was unrelated to the intussusception. Thus, the nonspecific right lower quadrant abdominal pain was the only symptom this patient presented with.

Various studies list nonspecific abdominal pain as the most frequently cited symptom, with prevalence ranging from 76.5% to 93.8% in the studied populations [6,7]. As abdominal pain alone is not diagnostic of intussusception, individuals presenting to the emergency department with this finding should undergo imaging to reach the correct diagnosis.

CT is regarded as the most accurate imaging modality for diagnosing intussusceptions in adults. Prior studies have found abdominal CT to accurately diagnose intussusception in 82-91% of cases [6,8]. In the aforementioned case, an abdominal CT successfully identified the patient’s intussusception but did not locate the lead point.

Surgical methods utilized in the treatment of adult intussusception include colon resection, hemicolectomy, and sigmoidectomy [8]. Preoperative reduction of the intussusception is not recommended due to the risk of seeding the cancer. As intussusception in adults is a rare phenomenon, reoccurrence rates after resection of the intussusception are not described in the literature. In a retrospective review of 41 cases of adult intussusception, Wang et al. found intussusception reoccurrence in a single patient [9]. This patient had several bowel adenomas acting as additional lead points. Additionally, a case report noted a reoccurrence of adult intussusception in a patient who underwent surgical reduction without resection [10]. Surgical resection of the intussusception, including the lead point, should prevent reoccurrence. Future research is needed to clarify reoccurrence rates of adult intussusception after various surgical procedures.

## Conclusions

Intussusception is an uncommon yet serious condition in adults due to its association with neoplasms. Its presentation varies from nonspecific abdominal pain to symptoms of bowel obstruction. Adult intussusception should be considered a differential for abdominal pain, specifically if the pain persists for weeks. CT is considered the optimal diagnostic tool for identifying intussusceptions, though a lead point is not always discovered. Early diagnosis using abdominal CT along with timely surgical resection prevents complications and improves patient outcomes. This report aims to describe a presentation of an adult intussusception due to colonic adenocarcinoma and increase clinical recognition of the condition among adults presenting with nonspecific abdominal symptoms.

## References

[1] Marinis A, Yiallourou A, Samanides L, Dafnios N, Anastasopoulos G, Vassiliou I, Theodosopoulos T (2009). Intussusception of the bowel in adults: a review. World J Gastroenterol.

[2] Kim KH, Namgung H, Park DG (2014). Adult intussusceptions: preoperative predictive factors for malignant lead point. Ann Surg Treat Res.

[3] Lu T, Chng YM (2015). Adult intussusception. Perm J.

[4] Brayton D, Norris WJ (1954). Intussusception in adults. Am J Surg.

[5] Kim KH (2021). Intussusception in adults: a retrospective review from a single institution. Open Access Emerg Med.

[6] Gomes A, Sousa M, Pignatelli N, Nunes V (2013). Adult intussusception: a single-center 10-year experience. Eur Surg.

[7] Sun M, Li Z, Shu Z, Wu Q, Liu X (2022). Adult intussusception: a challenge to laparoscopic surgery?. PeerJ.

[8] Tarchouli M, Ait Ali A (2021). Adult intussusception: an uncommon condition and challenging management. Visc Med.

[9] Wang N, Cui XY, Liu Y, Long J, Xu YH, Guo RX, Guo KJ (2009). Adult intussusception: a retrospective review of 41 cases. World J Gastroenterol.

[10] Akashige T, Sato K, Odajima H, Yamazaki S (2020). A case report of recurrent intussusception caused by small bowel lymphangioma in an adult. Int J Surg Case Rep.

